# Female urethral diverticulum: cases report and literature

**DOI:** 10.1186/1750-1164-8-1

**Published:** 2014-02-15

**Authors:** Omar Riyach, Mustapha Ahsaini, Mohammed Fadl Tazi, Soufiane Mellas, Roos Stuurman-Wieringa, Abdelhak Khallouk, Mohammed Jamal El Fassi, Moulay Hassan Farih

**Affiliations:** 1 Department of Urology, University Hospital Center Hassan II-FES, Fès, Morocco; 2 Department of Anatomy, Faculty of Medicine and Pharmacy of FES, Fès, Morocco; 3 Department of Urology, Reinier de Graaf Gasthuis, P.O. Box 5011, 2600, GA Delft, The Netherlands

**Keywords:** Urethra, Diverticulum, Woman, Diverticulectomy, Endovaginal approach

## Abstract

**Introduction:**

A female urethral diverticulum is an uncommon pathologic entity. It can manifest with a variety of symptoms involving the lower urinary tract. Our objective is to describe the various aspects of the diverticulum of the female urethra such as etiology, diagnosis and treatment.

**Cases presentation:**

We report five female patients, without prior medical history. They had different symptoms: dysuria in four cases, recurrent urinary tract infection in three cases, stress incontinence in two cases and hematuria in two cases. All patients had dyspareunia. The physical exams found renitent mass located in the endovaginal side of urethra which drained pus in two cases. Urethrocystography found a diverticulum of urethra in all cases. Our five patients underwent diverticulotomy by endovaginal approach. The course after surgical treatment was favorable. The urinary catheter was withdrawn after ten days. Some recurrent symptoms were reported.

**Conclusion:**

Evaluation of recurrent urinary complaints in young women can lead to the finding of a diverticulum of urethra. Urethrocystography can reveal this entity. Diverticulectomy by endovaginal approach is the best choice for treatment.

## Introduction

Originally described by William Hey in 1786, the urethral diverticulum in women is a rare disease. Its diagnosis is based on clinical presentation and examination with additional cystoscopy and urethral opacification. Treatment is considered strictly surgical.

Through presenting the five cases of female urethral diverticulum and literature review, we reviewed the diagnostic, therapeutic and evolutive aspects of this pathology.

### Case presentation

From 2000 to 2012, five women (mean age 40 years, range 23–58) with an urethral diverticula were diagnosed and treated at our institution. The diagnosis was based on reported symptoms, physical examination (Table 1), retrograde and voiding cysto- urethrography.

**Table 1:** Summary of the clinical signs in reported patients

**Symptoms**	**Number of patients**
Dysuria	4
Vaginal mass	3
Recurrent urinary tract infections	3
Dyspareunia	5
Stress or urge incontinence	2
Haematuria	2
Discharge of pus per urethra	2

### Case presentation 1

A 37-year-old Berber woman presented with a three year history of progressive urinary symptoms consisting of dysuria, urinary frequency, recurrent urinary tract infections as well as dyspareunia.

Clinical examination revealed a clean perineum, a tender cystic lesion located in the anterior vaginal wall with purulent vaginal discharge on palpation.

**Figure 1:**
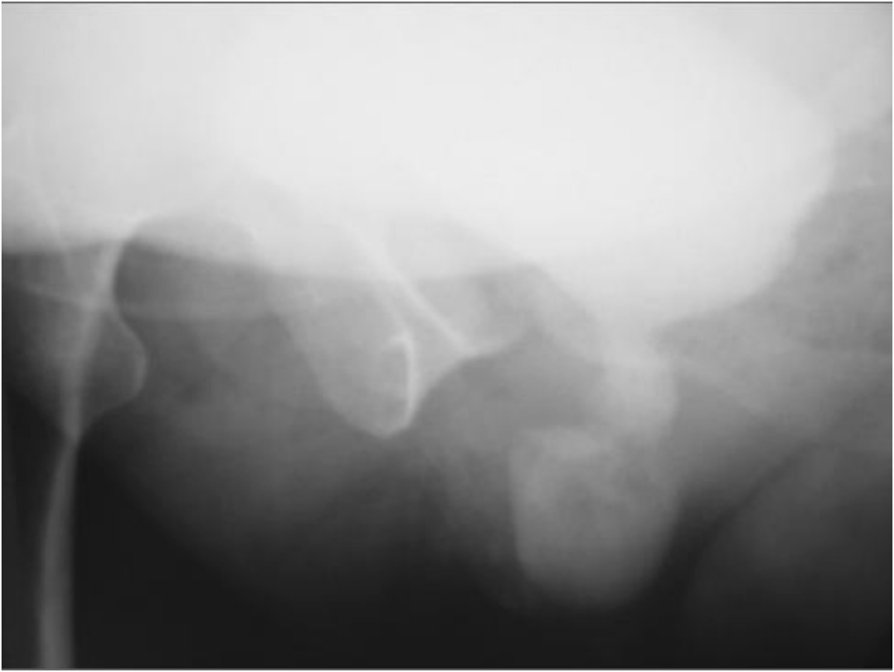
Voiding cystourethrogram shows a diverticulum filled with contrast agent at the level of the midurethra.

The retrograde and voiding urethrocystography revealed a urethral diverticulum at the middle third of the urethra (Figure 1).

The patient underwent transvaginal diverticulectomy by incision of the anterior vaginal wall and excision of the diverticulum after locating the connection to the urethra. An urethrovesical catheter was left 10 days. Follow-up examination after a few months showed no abnormality.

### Case presentation 2

A 32 years old Arab mother, with no previous gynaecological history, consulted our clinic with multiple urinary complaints: dysuria, haematuria, recurrent urinary tract infections and dyspareunia. The physical examination revealed a renitent mass filled with pus at the distal segment of the urethra.

**Figure 2:**
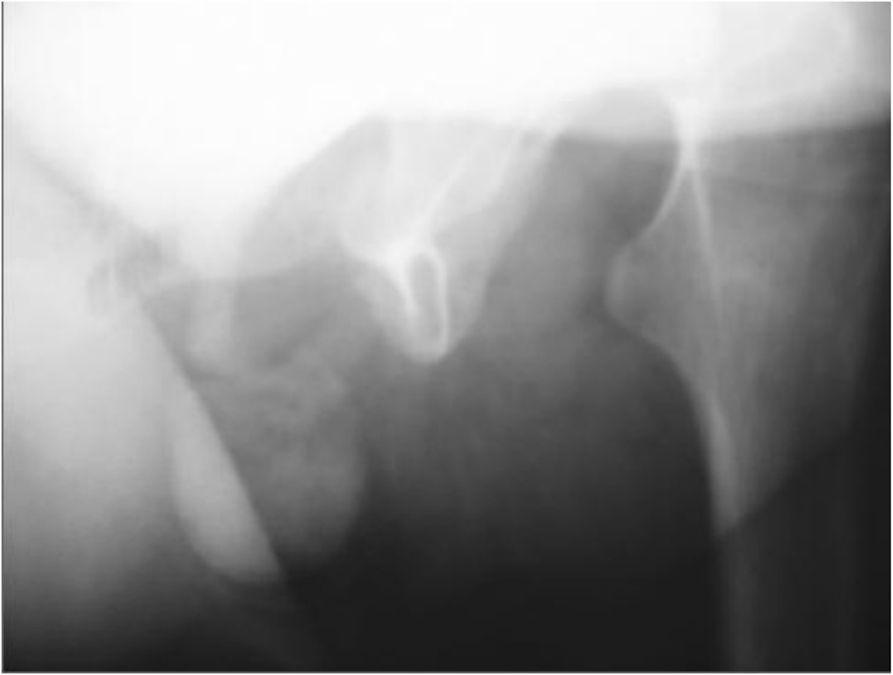
Urethrovesical opacification reveals a diverticulum of the urethra.

The patient underwent a retrograde and voiding urethrocystography that revealed a compound diverticulum of the urethra (Figure 2).

A transvaginal diverticulectomy was performed using the previously described procedure. The urethral catheter was left 10 days. Recurring dyspareunia was reported at follow-up examinations.

### Case presentation 3

A 40 years old Berber woman, with three homebirth children, presented with a four months history of stress urinary incontinence, recurrent urinary infections, dyspareunia and intermittent drainage of pus from the urethral meatus. Physical examination showed renitent mass located in the endovaginal side of urethra that drained pus.

The patient underwent a retrograde and voiding urethrocystography revealing a diverticulum at the proximal third of urethra.

A transvaginal diverticulectomy was performed by opening, dissecting and excising the diverticulum with no postoperative complications. The urethrovesical catheter was removed 10 days later. Recurring urinary tract infection was reported at follow up examination.

### Case presentation 4

A 23-year-old Berber woman presented with history of dysuria, urinary frequency, urge incontinence and dyspareunia.

Clinical examination revealed a clean perineum, and renitent vaginal mass with no inflammatory aspect.

The retrograde and voiding urethrocystography revealed a septed diverticulum at the distal third of urethra.

A transvaginal diverticulectomy was performed after incision of the anterior vaginal wall and excision of the diverticulum after locating the connection to the urethra. The urethrovesical catheter was left for 10 days. Follow-up examinations showed recurrent stress incontinence six months after surgery.

### Case presentation 5

A 58 years old woman married mother with a salpingectomy as gynecological antecedent consulted our clinic for urinary complaints consisting of haematuria, dysuria, and dyspareunia.

The physical examination was normal.

The patient underwent a retrograde and voiding urethrocystography revealing two diverticula at the proximal and distal third of the urethra.

The patient underwent a transvaginal diverticulectomy by opening, dissecting, and excising the diverticulum. The urethral catheter was left 10 days with no abnormality at follow-up examinations.

## Discussion

What is the origin of the Female Urethral Diverticulum? Is it congenital, iatrogenic, traumatic or infectious [1-5]?

The search for the origin of a diverticulum of the female urethra arouses from the passion of understanding its pathogenesis and many studies have been initiated to answer this question.

The congenital hypothesis is highlighted if the diagnosis is made in girls or in the neonatal period where the diverticulum is usually located on the roof of the urethra [6]. The diverticulum is considered to develop from embryonic remnants (Gartner’s duct).

But the Routh’s [7] hypothesis is more supported. It highlights the role of iterative infection and obstruction of periuretral glands. This leads to the cysts and then diverticulum formation by breaking and spilling their contents into the urethral lumen giving way to a pocket that is covered with epithelial component.

A well conducted interview and physical examination are able to raise the hypothesis of the diagnosis of urethral diverticulum. The classic triad (dribbling, dyspareunia, dysuria) [6,8-10], recurrent urinary tract infections and irrigative signs are all very supportive signs of urethral diverticulum [1].

Investigations include, as we report in our observations, the retrograde and voiding urethrocystography but also retrograde urethrocystography with positive pressure tests. This has the advantage of being easy accessible and allowing the urologist to participate in the interpretation. Other diagnostic techniques require specific hardware and remain operator dependent, such as MRI with endorectal coil [11-13]. This investigation is good to detect and locate small diverticulum with a sensitivity between 70 and 100%, but a limited specificity.

Ultrasound is a non-invasive examination with no risk of allergy or risk of radiation and some studies have shown sensitivity between 86 and 100% [14,15] in the diagnosis of diverticulum. However ultrasound remains to be an operator-dependent investigation which can miss small diverticula.

The additional advantage of an urethrocystoscopy is the possibility of the diverticular neck localization and its urethral topography [1,6].

Clinical presentation, imaging and urethrocystoscopy can confirm the diagnosis of urethral diverticulum and eliminate other diseases that can be confused with the diverticulum such as Cartner duct cysts, peri-meatiques lesions and Skene para-uretrales glands abscesses.

The treatment is strictly surgical consisting of a diverticulectomy [16-18], by transvaginal approach. Some surgical teams advise a prone position [1,16,19,20] with the argument that it allows better exposure of the diverticulum. However a supine position, which we adapt for our patients, allows a better exposure of distal lesions and allows an easy and complete excision of the diverticulum. We can consecutively treat an associated stress urinary incontinence by performing a colposuspension (anim). This position has also the undeniable advantage of a locoregional anesthesia [1].

By opting for a U incision, which is our personal choice and recommended by Raz [5], our objective was to reduce the risk of overlapping suture lines, fistula and post-operative stenosis.

The type of incision, the vaginal transverse incision, arcuate or inverted U, remains a choice of the surgical team without major outcome changes. The best way to close the diverticular collar is in three layers: the urethra, the periurethral fascia and vaginal wall. This technique reduces significantly [1] the risk of fistula or postoperative incontinence.

For urine drainage, we opted for the urethral catheter for 10 days but some authors recommend cystostomy alone [1] or the combination of a cystostomy and urethral catheter [6].

Like any surgery, treatment of an urethral diverticulum has complications consisting of fistula, urethral stenosis and urinary incontinence or lower urinary tract sympoms with a frequency reported between 3 and 10% [8,21]. Recurrence is rare [4,6], the literature highlights some predisposing factors as: urethral infection, local inflammation and urine drainage problems [5].

The surgical diverticulectomy is the gold standard in the treatment of this disease however there are some other therapeutic treatments such as vaginal marsupialization of Spence and Duckett and endoscopic incision of the diverticular neck, technique. Thess involve a significant risk of postoperative incontinence and are restricted to the distal diverticula.

## Conclusion

A urethral diverticulum is a rare disease with poorly understood pathogenesis. Clinical presentation with diagnostic, retrograde and voiding urethrocystography can confirm the diagnosis. The treatment is purely surgical.

### Consent

Written informed consent was obtained from all patients (case 1,2,3,4,5) for publication of this manuscript and accompanying images.

## Competing interests

Authors declare that they have no competing interests.

## Authors’ contributions

OR, MA: the principal authors, major contributions in writing the manuscript. MFT, SM, RSW , AK, MJF, MHF: analysed and interpreted the patient data and the reviews of the literature. All authors read and approved the final manuscript.
